# Traumatic Extremity Amputations in Children: Causes and Outcomes Based on a Regional Experience

**DOI:** 10.5811/westjem.65072

**Published:** 2026-08-29

**Authors:** Joyce J. L. H. McRae, Joseph Whitaker, Rebecca Dolman, Rosemary Vannix, Liang Ji, Andrei Radulescu, Donald Moores

**Affiliations:** * Loma Linda University Health, Department of General Surgery, Division of Pediatric Surgery, Loma Linda, California; † Loma Linda University Health, Department of General Surgery, Loma Linda, California; ‡ Loma Linda University School of Public Health, Department of Epidemiology, Biostatistics and Population Medicine, Loma Linda, California

## Abstract

**Introduction:**

Pediatric traumatic extremity or digit amputations are preventable causes of morbidity. The object of this study was to evaluate the etiology and outcomes of these injuries in our region. We used the Childhood Opportunity Index to explore the role of social determinants of health.

**Methods:**

We performed a retrospective cohort study at an academic children’s hospital on all pediatric patients younger than 18 years who presented with a traumatic digit or extremity amputation between January 1, 2014, to July 31, 2024. Metrics such as patient demographics, injury severity, prehospital course, hospital events and follow-up were abstracted. The Childhood Opportunity Index assigns a 0–100 composite score of region-based childhood opportunity determined from home ZIP codes. Our primary outcome measure was mechanism of injury. Secondary outcomes included length of stay, need for initial surgical intervention, subsequent operations, or unplanned readmissions.

**Results:**

A total of 73 patients presented with a traumatic amputation of a digit or extremity. The median age was 12.7. The top three causes of amputations were all-terrain vehicle (ATV) crashes, fireworks, and entrapment in an object. All-terrain vehicle crashes were the most common cause in all age groups from 5–17, while caught in an object was the most common for ages 0–4. The median Injury Severity Score was 5 (IQR 7). The three most common sites of amputation were right index finger (21), right long finger (17), and left long finger (15). Thirty-three children (45.2%) had more than one amputation. The highest number of amputations in a single patient was 10. All patients underwent a surgical intervention, and there were no mortalities. The median Childhood Opportunity Index score of the cohort was low at 19 (IQR 33), with 73% (n = 53) of the cohort originating from a very low (n = 37) or low (n = 16) index region. A rising index score was weakly associated with a decrease in total number of amputations (ρ = −0.25 [CI, −0.45 to −0.02, *P* = .04]).

**Conclusion:**

A. total of 73 children in our region suffered a traumatic digit or extremity amputation, mostly due to ATV crashes and fireworks. Most patients originated from a neighborhood of very low or low Childhood Opportunity Index scores. Increased public awareness regarding the danger of ATVs in regions with lower index scores may prevent these injuries. Clear, targeted policies toward ATV usage in childhood are needed.

## INTRODUCTION

Traumatic amputations in children are a devastating source of morbidity and are assumed to be caused by preventable events. In a recent review of the National Electronic Injury Surveillance System from 2012 to 2021, it was estimated that the most common age demographic to suffer from a near or complete traumatic amputation was the 0–5 year-old age group.^1^ Of all the estimated pediatric patents included, fingers were the most common body part affected, and doors were the most common consumer product to cause injury in children.^1^ However, there has been recent concern that not all amputation injuries in children should be presumed to be accidental. A review of the New York Statewide Planning and Research Cooperative System from 2004 to 2013 suggested that pediatric patients presenting with diagnostic code for abuse were significantly more likely to have a fingertip injury than those who did not.^2^

The cost of an amputation in a child involves more than just the physical losses and has reverberations in healthcare costs and family dynamics. Vakhshori et al found that, in the United States, there was a total national healthcare use cost of $166 million for 6,130 pediatric traumatic upper extremity injuries from 1997 to 2012.^3^ Furthermore, in a survey of caregivers of children with limb loss, there was a notable increase in missed school for the child, and half of caregivers needed to adjust their work participation due to the injury.^4^

This study was prompted by a perceived increase in the case burden of these devastating injuries presenting to our hospital and a desire to understand the cause of these events. Our primary aim was to evaluate the mechanism of these events by age group, with secondary aims including understanding overall injury severity in this population and analyzing the relationship of social determinants of health via the Childhood Opportunity Index with these injuries. The Childhood Opportunity Index is a composite indicator revealing the impact of a child’s neighborhood on their overall opportunities in life; it encompasses numerous metrics including economic resources, environmental impact, and community social capital.^5^ This indicator has been used in previous trauma literature as a surrogate marker of social determinants of health to evaluate the impact of a child’s home origin on trauma outcomes.^6^ A lower Childhood Opportunity Index has been shown to be associated with violent mechanisms of injury, but further research is needed in other geographical regions to continue to validate its role in addressing unmet health-related social needs.^7^

We hypothesized that the primary cause of traumatic extremity or digit amputations in children in our region would be due to motor vehicle collisions (MVC) and that most patients would come from a background with a lower Childhood Opportunity Index. Our children’s hospital is located next to a major interstate freeway and is surrounded by a region with poor public transport systems. This, coupled with a high burden of MVCs presenting to our children’s hospital, prompted our hypothesis. The objective of this study was to determine the number, mechanism, and clinical impact of traumatic extremity and digit injury amputations to our Level I pediatric trauma center over time.

**Population Health Research Capsule**

What do we already know about this issue?

*Traumatic amputations in children cause devastating morbidity and high healthcare costs*.

What was the research question?

*Our goal was to understand the rates, severity, and causes of traumatic extremity and digit amputations in southeast California*.

What was the major finding of the study?

*Seventy-three children suffered a traumatic extremity/digit amputation with nearly 1/3 caused by all-terrain vehicle crashes*.

How does this improve population health?

*Causes of preventable injury need to be delineated to improve awareness and advocacy*.

## METHODS

### Study Design and Patient Population

After institutional review board (IRB) approval (#5240372), a retrospective cohort study was initiated. We performed a review of the trauma registry at an academic children’s hospital with Level I pediatric and adult trauma designation. The institutional trauma database includes all patients with a traumatic injury who were admitted or received a trauma team activation. It does not capture patients who were treated and discharged from the emergency department (ED) without a trauma team activation. Inclusion criteria of the study included all patients younger than 18 years who presented with a traumatic digit or extremity amputation between January 1, 2014, to July 31, 2024. Amputations were initially identified by *International Classification of Diseases*, 9^th^ and. 10^th^ revisions, (ICD 9 and 10) codes provided in the trauma registry. Exclusion criteria included any patient over 18 years of age and the diagnosis of an incomplete amputation or an amputation of a nonextremity body part.

Data abstracted from the trauma database included patient origin, demographics, injury mechanism, outside hospital course, ED course and discharge details. Additional information was abstracted from the institutional electronic health record including location of amputation, operative details, hospital course, complications, and follow=up. Amputations were classified by location. Any severing amputation involving a hand or foot phalangeal joint, distal metacarpal or distal metatarsal were considered digit amputations. Amputations proximal to the metacarpal or metatarsal joints, or through the wrist, upper extremity, mid foot or lower extremity were considered extremity amputations. A total of 73 children were identified during the study period.

### Use of Childhood Opportunity Index

The Childhood Opportunity Index version 3.0 is a composite index of 44 indicators that provides a ZIP code or US Census-based numerical assignment to describe the overall access to opportunities that a child residing in that neighborhood has.^8^ In version 3.0, numerical assignments are given based on quintiles from 1–100.^8^ Additionally, in version 3.0, scores were derived for years 2012 to 2021.^8^ In this analysis, scores were assigned based on ZIP code of patient’s home address and corresponded to year of injury, except for years 2022 to 2024, which were based on the scores from 2021 due to lack of Childhood Opportunity Index data from those years at time of research study. Then, patients were assigned into the opportunity categories. For a subanalysis, we combined moderate, high and very high into one category.

### Statistical Analysis and Method Criterion

We used descriptive statistics for analyzing demographics and clinical characteristics using Excel (Microsoft Corporation). Additional analyses were conducted to assess correlation or differences between demographic and clinical covariates and three Childhood Opportunity Index subgroups, including the Spearman correlation and Wilcoxon rank-sum test. Statistical significance was denoted by *P* value < .05. We used SAS for analysis (SAS Institute, Inc). The Strengthening the Reporting of Observational Studies in Epidemiology (STROBE) checklist for cohort studies was used to ensure proper information reporting. We followed the method criteria outlined by Worster and Bledsoe: abstractor training; defined case selection criteria; defined variables; consistent abstraction form; monitored performance; interobserver reliability discussion; medical record identification; described sampling method; and IRB approval.^9^

## RESULTS

### Demographics, Childhood Opportunity Index, and Interventions

Of the 73 children identified in the study, the median age was 12.7 years (range 1.2 to 17.9 years). Most patients injured were male (71%) and identified as Hispanic (59%). In 2021, 58.2% of children in our institution’s county identified as Hispanic or Latino.^10^ The median body mass index (BMI) for the entire cohort was 19.7 (IQR 8.5). The median Childhood Opportunity Index of the cohort was low at 19 (IQR 33). When the cohort was divided into five Childhood Opportunity Index categories, just over half the cohort was shown to live in ZIP codes identified as very low opportunity neighborhoods (51%). Most patients originated from a neighborhood of very low or low opportunity (73%) while the minority (27%) of patients lived in a ZIP code identified as providing moderate, high, or very high opportunity. These results are relatively similar to the overall trauma population presenting to our institution. In 2021, 75.5% of pediatric patients presented from a neighborhood of very low (46.6%) or low opportunity (28.9%) and 24.5% from a neighborhood of moderate (14.6%), high (6.8%) or very high (3.1%) opportunity.

All patients required a surgical intervention, and 14% of patients were fitted or planned to be fitted for a prosthesis after surgical intervention. Nonextremity surgical interventions were required in 11% of patients. Demographics of the cohort are summarized in Table 1.

### Severity and Rates of Injuries

The overall rate of injury presentation has fluctuated over time with an overall positive trajectory, ranging from four to nine patients per year. The number of patients treated during the first half of 2024 (n = 8) nearly met the highest rate overall rate of injured patients seen in 2018 (n = 9). For the overall cohort, the median Injury Severity Score (ISS) was 5 (IQR 7). Median length of stay (LOS) for the cohort was 2.9 days. The average LOS was 7.3 days, likely skewed by one patient with a maximum stay of 129.9 days. During our study period, 9,657 pediatric patients with a traumatic injury were admitted or received a trauma team activation. The percentage of traumatic amputations in this cohort was rare at 0.76%.

### Mechanism of Injury

The most common mechanism of a traumatic amputation in this cohort was all-terrain vehicle (ATV) crash (or four-wheeler crash), followed by fireworks and an appendage being entrapped or caught in an object such as a door or fence. The next most common causes were power tool accidents (eg, table or chop saw), falling objects, industrialized machinery equipment accidents (eg, a hydraulic wood splitter or conveyor belt) and landscaping equipment (eg, a hedge trimmer or clippers). Less common causes were due to bicycle, motorcycle or dirt bike accidents, boating, rodeo roping competition, or animal bite. Surprisingly, MVC was tied for the seventh most common cause of injury. Figure 1 outlines all mechanisms of traumatic amputations in our cohort.

### Injury Mechanism by Age Group

To better understand specific risk factors of these injuries, the cohort was split by age groups. The injury causes by age group were surprisingly similar. For patients in age group 0–4, an appendage becoming entrapped in an object was the most common cause. However, for all three additional age groups (5–9, 10–13 and 14–17) the most common cause of amputation was due to ATV crashes, with fireworks tying for first in the 10–13 age group. Fireworks were also the second leading cause in age groups 5–9 and 14–17. The distribution of mechanism of injury by age group is displayed in Figure 2.

### Sites of Amputation

Site of amputation was determined by chart review to ascertain the approximate location of amputation, and each appendage lost was counted separately. Most injuries occurred in the hands, with fingers being the most affected. The five most frequently amputated appendages were right index finger (n = 21), right long finger (n = 17), left long finger (n = 15), left ring finger (n = 14), and left small finger (n = 11). Patient handedness was not consistently reported. Figure 3 outlines the number of amputations by site of amputation. Thirty-three patients had more than one site of amputation. The highest number of amputation sites was 10 in a patient who lost all fingers due to their injury. Fifteen extremities were lost ranging from the wrist to the upper leg at the femur. The mechanisms of complete extremity amputations were ATV crashes (n = 5), fireworks (n = 4), automotive versus pedestrian accidents (n = 3), motor vehicle collision (n = 1), motorcycle collision (n = 1) and animal bite (n = 1). The total number of amputated digits and extremities for the cohort were 146.

### Impact of Childhood Opportunity Index on Cohort Amputation Rates and Outcomes

To understand whether a child’s Childhood Opportunity Index impacted the severity, rate, or outcome of a traumatic amputation, we conducted a subanalysis (Table 2). Due to a low number of total patients in the moderate opportunity, high opportunity and very high opportunity cohorts, these three were combined into one group in the analysis (n = 19). One patient was from outside the country and did not have an assigned Childhood Opportunity Index and was excluded from this analysis. The only relationships that showed statistical significance was that rising Childhood Opportunity Index (increased opportunities) was weakly and negatively correlated with the total number amputations a patient suffered (ρ = −0.25; *P* = .04). There was no correlation between Childhood Opportunity Index and ISS, requiring nonextremity surgery for traumatic injuries, LOS in days, whether a prosthetic was planned or fitted, the need for a second surgery, unplanned readmission, unplanned ED visit, or amputation-related surgical complications.

## DISCUSSION

Traumatic extremity and digit amputations in children in our region were primarily caused by ATV crashes and fireworks in nearly all age groups. The causes of these injuries in our cohort are preventable. Educating parents on recreational vehicle safety and avoiding celebratory explosive devices is crucial to preventing these devastating injuries. In the United States, traumatic amputations in children have been associated with lawnmower accidents, specifically riding motors instead of push mowers, farming accidents, and animal encounters.^11^–^13^ Internationally, documented causes have included household meat grinders in Turkey, MVCs in England, or doors in Greece.^14^–^16^ study from Bertani et al describes their experience with 89 children who suffered war-related extremity injuries in Afghanistan.^17^ Explosive devices were the highest cause of injuries (79%), and nearly 5% of their cohort died from the injuries received.^17^

A large review conducted in 2010 of 956 U.S.-based cases noted that digit amputations accounted for the majority of amputations (64%), similar to our patient population.^18^ In 2017, a review of the National Trauma Data bank (NTDB) from 2007 to 2011 was conducted, analyzing 2,238 pediatric patients for trends in causes of amputations.^19^ The authors reported that common amputation locations were fingers (54%) and toes (20%) with most amputations occurring in patients younger than 5 years and older than 15 years.^19^ The former national review concluded that pediatric traumatic amputations created a large healthcare cost burden and prevention efforts should be prioritized to reduce these injuries and their associated healthcare costs.^18^ The latter review noted that despite perceived increases in public awareness regarding the impact of specific causes such as lawnmowers current prevention efforts were inadequate.^19^ Landscaping equipment was not a major cause of amputation in our region, potentially signaling that in the past decade, targeted prevention strategies for lawnmowers was successful, but this will need verification from larger national studies. The NTDB review noted that injuries were more common in younger and older children, but our cohort was skewed toward increased teenage population (14–17 years old).^19^ It is possible that this was impacted by our regional referral pattern with severe injuries transferred in and less severe injuries managed locally.

In Vakhshori et al’s review of the Healthcare Cost and Utilization Project (HCUP) Kids’ Inpatient Database from 1997 to 2012, the most common causes of traumatic upper extremity amputations, if known, were lawn mower/machinery accidents and being caught in an object, with fireworks rounding out the top five mechanisms.^3^ There is no description of ATV crashes, but MVCs were one of the main causes of major amputations above the elbow, with increasing rates over the study period while most other causes decreased in incidence.^3^ Our patient cohort reflected overall similar mechanisms but a differing distribution of primary causes. This national sample had a similar overall median age of 12.1 compared to 12.7 in our cohort, signaling that the burden of this risk of amputation is in older elementary school children (aged 9–11 years).^3^ The large cohort had an average LOS of 3.4 days, compared to our study’s average of 7.3 days.^3^ Our median LOS was 2.9 days, reflecting that our average was skewed by multiple high outliers demonstrating the range of impact that these injuries can have on healthcare burden. Although we did not review cost of treatment in our population, the mean hospital charge for the HCUP cohort was nearly $29,000 and increased with age.^3^ This may reflect the higher burden of injury in older patients.

In our patient population, we were surprised to find that our three main regional culprits were ATV crashes, fireworks, and a digit being caught in an object, the latter a similar cause as found in the Greek cohort. We hypothesized that the cause of amputations would be primarily due to MVCs due to our proximity with multiple major interstate highways and our location in an urban environment with a rural catchment area. Two patients suffered from a traumatic amputation due to MVC, but ATV crashes were the clear cause of amputation-related injuries, spanning multiple age groups. Recent studies are raising the alarm of the danger of these recreational vehicles in children. Willis et al reported nearly 500 cases of ATV-related injuries at their Level I pediatric trauma center, calling for increased prevention education.^20^ Concerns regarding the impact of off-road vehicle (ORV) crashes in our region are rising. Recent work from Kenney et al described the patterns of injuries and impact of social vulnerability on ORV crashes in the adult trauma population in our region.^21^ Over a six-year period, 574 adult patients were treated at our institution for ORV crashes with the majority originating from a region of high social vulnerability.^21^ It is possible that future analyses of pediatric ORV crashes in our region will demonstrate a similar pattern of higher vulnerability groups being disproportionally impacted.

The primary causes of traumatic extremity and digit amputations are accidental, as demonstrated in our study, but nonaccidental trauma should be considered by clinicians caring for these patients.^2^ The management of these injuries may vary depending on location of care. In a review of the HCUP Project Kids’ Inpatient Database from 2000 to 2006 regarding pediatric finger injuries, Squitieri et al found that most children were treated at nonchildren’s hospitals but that there were notable disparities in the attempted reimplantation rates for Black, Hispanic, or uninsured patients.^22^ A similar paper using the same database queried it from 2000 to 2012 to understand disparities in those who underwent reimplantation for thumb and finger amputations finding that most were younger, female, and had private insurance.^23^

Our cohort did not have any documented nonaccidental trauma concerns. However, 50% of the cohort resided in a neighborhood with very low opportunity. Only 27% lived in a neighborhood that was considered to have moderate, high or very high childhood opportunity. In our subanalysis, we found that although this was a striking difference, the only outcome that correlated with the Childhood Opportunity Index grouping was a small increase in total number of amputation injuries. All other differences in ISS, LOS, unplanned readmissions, unplanned ED visits, or amputation-related surgical complications were not statistically significant between the groups. However, due to the strains linked to residing in a lower opportunity neighborhood, long-term outcomes including rehabilitation attendance should be assessed to identify areas in which these families can better be supported. Buncke et al noted in their review of pediatric mutilating hand injuries that conscientious, advocative, and consistent parental support was key for fostering the best rehabilitation outcomes.^24^

Targeted public health interventions are needed to address the causes of traumatic amputations in children in our region, particularly due to ATV crashes and fireworks. Awareness regarding the significant morbidity caused by recreational vehicles and fireworks is a first step.

## LIMITATIONS

There are limitations to this study due to its retrospective nature and regional referral patterns. Our trauma database does not include traumatic injuries that were exclusively treated in the ED without a trauma activation or admission; therefore, minor injuries may have been missed. There are some missing data points in the electronic health record, and multiple patients were lost to long-term follow-up, precluding the ability to analyze the long-term effects of these injuries. We were unable to ascertain handedness in most patients from medical records, limiting inferences based on side of injured upper extremity. Although our hospital receives many pediatric transfers for pediatric trauma continuation or pediatric hand specialization consultation, our regional sample is likely missing patients cared for primarily by rural hospitals, thus limiting our overall cohort size. Additionally, we do not have complete injury patterns of all trauma mechanisms, limiting the evaluation of incidence of these injuries. Furthermore, at the time of study, Childhood Opportunity Index data was not available for years 2022 to 2024; therefore, the index level assignments were extrapolated from 2021 data, which may impact the accuracy of comparisons of groups. The Childhood Opportunity Index category comparisons were exploratory in nature and not prescriptive. Further studies are needed to target the long-term implications of these injuries when obtained during childhood.

## CONCLUSION

A total of 73 children in our region suffered a traumatic digit or extremity amputation over 9.5 years. The two most common causes were ATV (or four-wheeler) crashes and fireworks. The most common location of amputation was the right index finger, right long finger, and left long finger. Most patients lived in a neighborhood with very low or low Childhood Opportunity Index scores. Increased targeted public awareness of the growing danger of ATV crashes and fireworks in our community may inform prevention of these life-altering injuries.

## Figures and Tables

**Figure 1:**
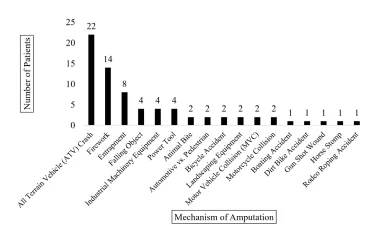
All mechanisms of traumatic extremity or digit amputations in pediatric cohort at single children’s hospital.

**Figure 2:**
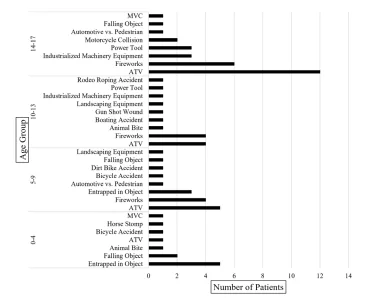
All mechanisms of traumatic extremity or digit amputations divided by age groups at a single children’s hospital. *ATV*, all-terrain vehicle Crash; *MVC*, motor vehicle collision.

**Figure 3:**
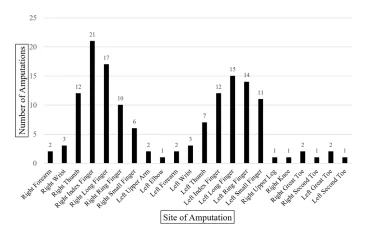
Number and site of extremity or digit amputation of entire pediatric cohort at a single children’s hospital.

**Table 1:** Demographics of 73 pediatric patients with traumatic extremity or digit amputations in a study evaluating the etiology and outcomes of these injuries using the Childhood Opportunity Index to explore the role of social determinants of health.

Age, median [IQR], years	12.7	[8.4]
Male Sex, n (%)	52	(71)
Race, n (%)		
Hispanic	43	(59)
White	23	(32)
Black	3	(4)
Asian	1	(1)
Other	3	(4)
Body mass index, median [IQR]	19.7	[8.5]
Childhood Opportunity Index score, median [IQR]	19	[33]
Childhood Opportunity Index categories, n (%)		
Very Low	37	(51)
Low	16	(22)
Moderate	9	(13)
High	5	(7)
Very High	5	(7)
Injury Severity Score, median [IQR]	5	[7]
Nonextremity surgical intervention, n (%)	8	(11)
Prosthesis planned or fitted, n [IQR]	10	[14]

*IQR*, interquartile range.

**Table 2:** Analysis of Childhood Opportunity Index of pediatric cohort demonstrating no correlation between lower index score and Injury Severity Score or intervention required.

	Very Low (n = 37)	Low (n = 16)	Moderate to Very High (n = 19)	*P* value	ρ [CI]
ISS, median [IQR]	5 [6]	8 [6.5]	5 [8]	.66^	0.05 [−0.18 to 0.28]
Total number of amputations, median	2	2	1	**.04** ^	−0.25 [−0.45 to −0.02]
Non-extremity surgical intervention, n (%)	5 (13.5%)	1 (6.3%)	2 (10.5%)	.62^	
Length of stay, median days	3.7	2.1	2.6	.27^	−0.13 [−0.35 to 0.10]
Second operation, n (%)	16 (43.2%)	8 (50%)	7 (36.8%)	.78*	
Unplanned readmission, n (%)	3 (8.1%)	1 (6.3%)	1 (5.3%)	.69*	
Unplanned ED visit, n (%)	4 (10.8%)	2 (12.5%)	0	.25*	
Surgical complications from amputation, n (%)	9 (34.2%)	3 (17.8%)	3 (15.8%)	.44*	
Prosthetic planned or fitted, n (%)	7 (18.9%)	2 (12.5%)	1 (5.3%)	.17*	

^ Spearman correlation.

* Wilcoxon rank-sum test.

*ED*, emergency department; *IQR*, interquartile range; *ISS*, Injury Severity Score.
